# Trialling a Large Language Model (ChatGPT) in General Practice With the Applied Knowledge Test: Observational Study Demonstrating Opportunities and Limitations in Primary Care

**DOI:** 10.2196/46599

**Published:** 2023-04-21

**Authors:** Arun James Thirunavukarasu, Refaat Hassan, Shathar Mahmood, Rohan Sanghera, Kara Barzangi, Mohanned El Mukashfi, Sachin Shah

**Affiliations:** 1 University of Cambridge School of Clinical Medicine Cambridge United Kingdom; 2 Attenborough Surgery Bushey Medical Centre Bushey United Kingdom

**Keywords:** ChatGPT, large language model, natural language processing, decision support techniques, artificial intelligence, AI, deep learning, primary care, general practice, family medicine, chatbot

## Abstract

**Background:**

Large language models exhibiting human-level performance in specialized tasks are emerging; examples include Generative Pretrained Transformer 3.5, which underlies the processing of ChatGPT. Rigorous trials are required to understand the capabilities of emerging technology, so that innovation can be directed to benefit patients and practitioners.

**Objective:**

Here, we evaluated the strengths and weaknesses of ChatGPT in primary care using the Membership of the Royal College of General Practitioners Applied Knowledge Test (AKT) as a medium.

**Methods:**

AKT questions were sourced from a web-based question bank and 2 AKT practice papers. In total, 674 unique AKT questions were inputted to ChatGPT, with the model’s answers recorded and compared to correct answers provided by the Royal College of General Practitioners. Each question was inputted twice in separate ChatGPT sessions, with answers on repeated trials compared to gauge consistency. Subject difficulty was gauged by referring to examiners’ reports from 2018 to 2022. Novel explanations from ChatGPT—defined as information provided that was not inputted within the question or multiple answer choices—were recorded. Performance was analyzed with respect to subject, difficulty, question source, and novel model outputs to explore ChatGPT’s strengths and weaknesses.

**Results:**

Average overall performance of ChatGPT was 60.17%, which is below the mean passing mark in the last 2 years (70.42%). Accuracy differed between sources (*P*=.04 and .06). ChatGPT’s performance varied with subject category (*P*=.02 and .02), but variation did not correlate with difficulty (Spearman ρ=–0.241 and –0.238; *P*=.19 and .20). The proclivity of ChatGPT to provide novel explanations did not affect accuracy (*P*>.99 and .23).

**Conclusions:**

Large language models are approaching human expert–level performance, although further development is required to match the performance of qualified primary care physicians in the AKT. Validated high-performance models may serve as assistants or autonomous clinical tools to ameliorate the general practice workforce crisis.

## Introduction

Deep learning is a form of artificial intelligence (AI), which facilitates the development of exquisitely organized processing within an artificial neural network architecture, composed of multiple layers of interlinked perceptron nodes [1]. During supervised training of these models, the nature and weighting of communicating links between perceptrons is tuned to optimize performance in a predefined task. While also applied to structured (tabulated) data, as with longer-established computational techniques, deep learning has enabled AI to work with unstructured inputs and outputs, such as images, videos, and sounds [1]. In recent years, natural language processing (NLP) has leveraged deep learning to extend the analytical and productive capability of computational models to unstructured language.

Generative Pretrained Transformer 3.5 (GPT-3.5) is a large language model (LLM), trained on a data set of over 400 billion words from articles, books, and other forms of media on the internet [2]. ChatGPT is a web-based chatbot that uses GPT-3.5 to directly answer users’ queries. Unlike most chatbots previously trialed in clinical settings, ChatGPT facilitates free-text input and spontaneous output, as opposed to manually designed finite-state inputs and outputs [3]. ChatGPT has already begun to be trialed in medical contexts and has garnered attention for attaining sufficient accuracy in medical licensing examinations to graduate as a doctor, with even better performance recorded since the release of GPT-4 as the application’s backend LLM [4-6]. As primary care struggles with poor recruitment, increasing workload, and early retirement [7-9], the introduction of autonomous decision aids and advisors may complement existing initiatives to improve the provision of general practitioners (GPs) [7,10]. Innovation in this sector would enable maximizing of the value provided by practicing GPs, likely benefiting deprived and rural areas—where fewer doctors serve the population—the most [11].

The Applied Knowledge Test (AKT) of the Membership of the Royal College of General Practitioners (RCGP) must be passed for GPs to complete their training in the United Kingdom. A total of 200 questions—mostly multiple choice but with occasional requirement to input numbers or select from a longer list of potential answers—must be answered in 190 minutes by candidates at a computer workstation. Questions test mostly clinical knowledge (80%), as well as evidence-based practice (10%) and primary care organizational and management skills (10%). All questions are designed to test higher-order reasoning rather than simple factual recall.

Before trials of clinical applications of NLP chatbots can be designed, the proposed purpose of applications such as ChatGPT must be established, requiring thorough investigation of their strengths and weaknesses. To evaluate the utility of ChatGPT in primary care settings, we used the AKT as an existing standard met by all UK GPs. The distinct sections of the AKT enabled the investigation of the opportunities afforded by ChatGPT (and LLMs more broadly), as well as the limitations of currently available technology. Through this work, we aimed to provide suggestions as to how clinical and computational research should proceed with the design and implementation of NLP chatbots, supported by empirical data.

## Methods

### Overview

AKT questions were sourced from the RCGP’s GP SelfTest platform [12], as well as 2 publicly available practice papers [13,14]. Twenty questions were extracted from each subject category on the GP SelfTest platform, and all questions were extracted from the practice papers. Two researchers matched the subject categories of the practice papers’ questions to those defined in GP SelfTest and in AKT examiners’ reports from 2018 to 2022, with disagreements resolved through discussion and arbitration by a third researcher. Questions and multiple answer choices were copied from these three sources for entry into ChatGPT. Questions with multiple parts were prepared as distinct entries. Questions requiring appraisal of non–plain text elements that could not be copied into ChatGPT were excluded from the study. Duplicate questions were identified by a single researcher and excluded from the study.

Every eligible question was inputted into ChatGPT (January 30, 2023, version; OpenAI) on 2 separate occasions between January 30 and February 9, 2023, in separate sessions to avoid the second trial from being influenced by previous dialogue. ChatGPT’s answer was recorded, and its whole reply to each question was recorded for further analysis. If ChatGPT failed to provide a definitive answer, the question was retrialed up to 3 times, after which ChatGPT’s answer was recorded as “null” if no answer was provided. Correct answers (ie, the “ground truth”) was defined as the answers provided by GP SelfTest and the practice papers—these were recorded for every eligible question. ChatGPT’s responses were screened for “novel explanations”—defined as any information provided that was not included in the question or multiple choice answers—by a single researcher.

The scores required to pass the AKT in every examination undertaken in the last 2 years were collected from RCGP examiners’ reports for the AKT between 2018 and 2022 [15]. Additionally, the number of recommendations of “room for improvement” for each subject category in the last 5 years were collected to use as a measure of “difficulty” in subsequent analysis.

ChatGPT’s answers in both trials were compared to the correct answers to gauge performance and were compared to recent pass marks to assess ChatGPT’s prospects of passing the AKT. ChatGPT’s answers were compared between the 2 trials to measure the consistency of its responses. Performance was analyzed with respect to difficulty, explanation novelty, source, and subject to explore the strengths and weaknesses of ChatGPT. Nonparametric statistical analysis was undertaken due to the nonrandom nature of question design and small number of questions in some subjects. Effect sizes were reported with 95% CI and *P* values, with statistical significance concluded where *P*<.05. Statistical analysis was conducted in R (version 4.1.2; R Foundation for Statistical Computing), and figures were produced using Affinity Designer (version 1.10.6; Serif Ltd).

### Ethics Approval

Ethics approval was not required for this study as human participants were not involved.

## Results

**Figure 1:**
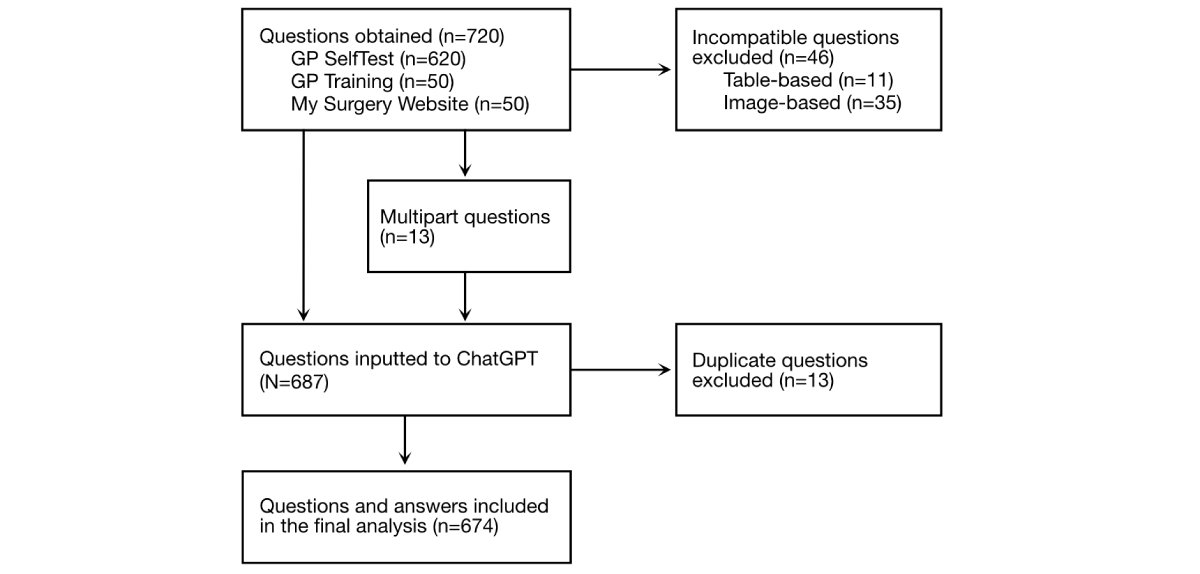
Flowchart illustrating how questions were sourced and processed before inputting into ChatGPT and extracting answers for further analysis. GP: general practitioner.

In total, 720 questions were identified, which increased to 733 questions after multipart questions were separated into distinct entries. In total, 674 unique questions were ultimately inputted into ChatGPT after duplicate and incompatible questions were excluded (Figure 1). Incompatibility was due to the question including an image in 35 cases and the inclusion of a table in 11 cases.

Exemplar questions and answers are depicted in Figure S1 in Multimedia Appendix 1. Overall performance was consistent: 59.94% (404/674) on the first run and 60.39% (407/674) on the second run. ChatGPT expressed uncertainty or did not provide an answer to repeated inquiry on 4 occasions in the first trial and on 6 occasions in the second trial, corresponding to 1.48% and 2.25% of incorrect answers, respectively. ChatGPT gave the same answer on both runs in response to 83.23% (561/674) of the questions, indicating variability in a significant proportion of cases. For reference, the average pass mark for the AKT in the last 2 years has been 70.42%, ranging from 69.00% to 71.00% [15]. Performance differed by question source (Table 1): variation was significant in the second (Fisher exact test, *P*=.04) but not the first (Fisher exact test, *P*=.06) trial. This indicates that question difficulty (for ChatGPT) differed between sources, although differences in performance were not large (Figure S2 in Multimedia Appendix 1).

**Table 1:** Overall performance of ChatGPT in both trials, stratified by question source.

Source			GP^a^ SelfTest [12]		My Surgery Website [13]		GP Training Schemes [14]
Questions, n			599		44		31
**Trial 1, n (%)**							
	Correct answers	368 (61.60)		23 (52.27)		13 (41.94)	
	Incorrect answers	231 (38.56)		21 (47.73)		18 (58.06)	
**Trial 2, n (%)**							
	Correct answers	372 (62.10)		21 (47.73)		14 (45.16)	
	Incorrect answers	227 (37.90)		23 (52.27)		17 (54.85)	

^a^GP: general practitioner.

**Figure 2:**
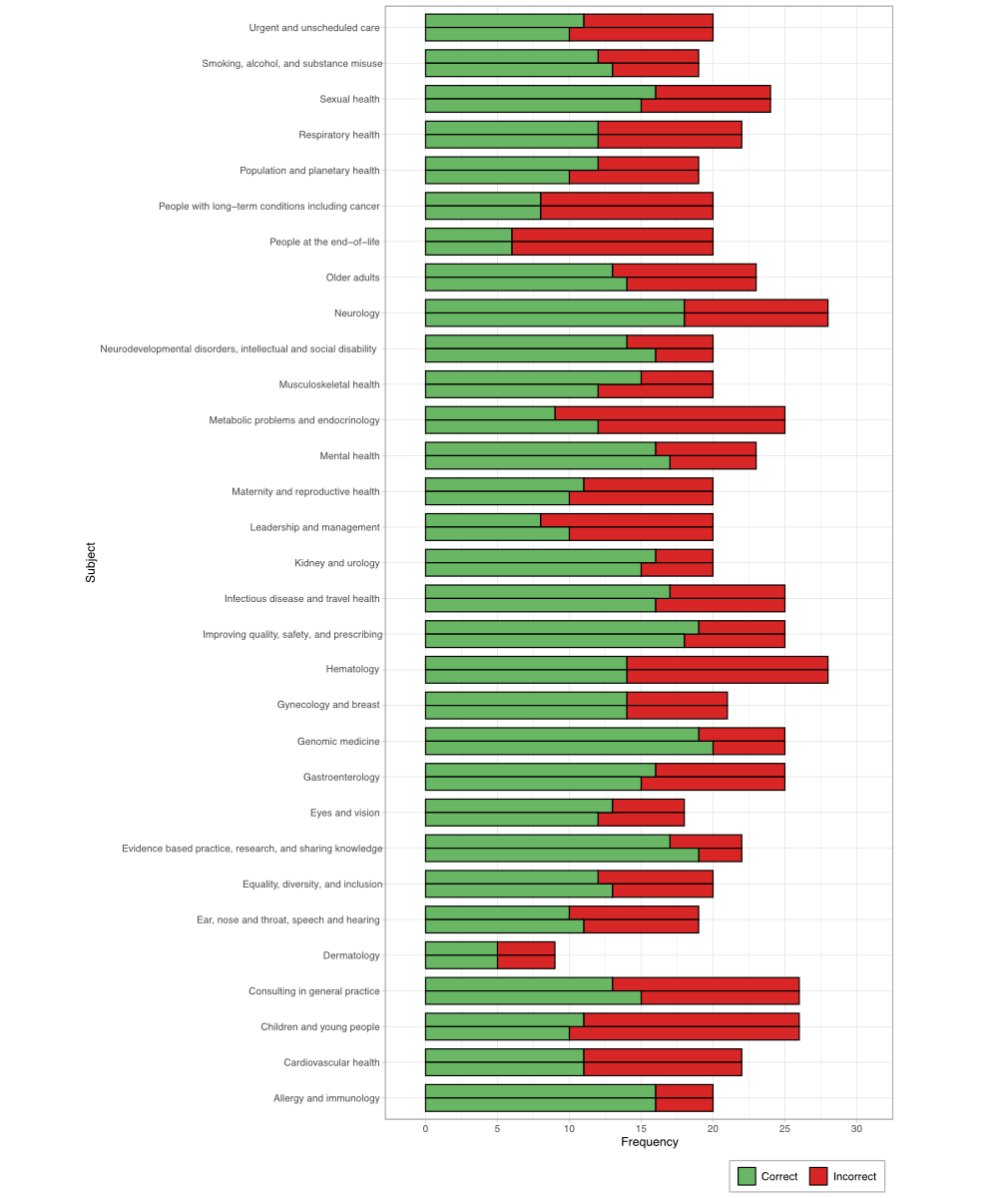
ChatGPT’s performance in 674 questions on the Membership of the Royal College of General Practitioners Applied Knowledge Test, stratified by subject category. The higher bar within each subject corresponds to the first trial; the lower bar corresponds to the second trial.

**Figure 3:**
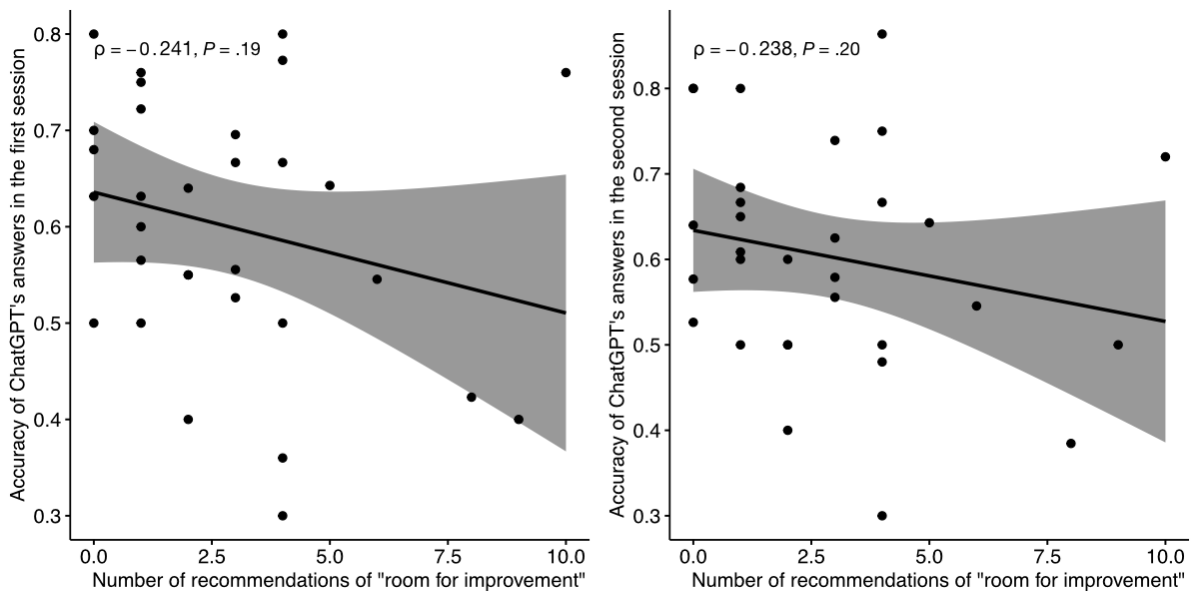
Correlation between ChatGPT performance and subject difficulty, expressed in terms of the Spearman rank correlation coefficient (ρ).

Performance was highly variable between subjects (Figure 2), with significant variation observed in the first (Fisher exact test estimated over 10^6^ iterations, *P*=.02) and second (Fisher exact test estimated over 10^6^ iterations, *P*=.02) trials. Subject variation did not correlate with the difficulty indicated by the frequency of recommendations of “room for improvement” by the RCGP (Spearman correlation coefficient for the first run [ρ]=–0.241, *P*=.19; Spearman ρ for the second run=–0.238, *P*=.20; Figure 3). Average accuracy over 75% was exhibited in 4 subjects: intellectual and social disability, kidney and urology, genomic medicine, and allergy and immunology (Table S1 in Multimedia Appendix 1). Accuracy under 50% on average was exhibited in 5 subjects: leadership and management, metabolic problems and endocrinology, children and young people, people with long-term conditions including cancer, and people at the end-of-life (Table S1 in Multimedia Appendix 1).

**Figure 4:**
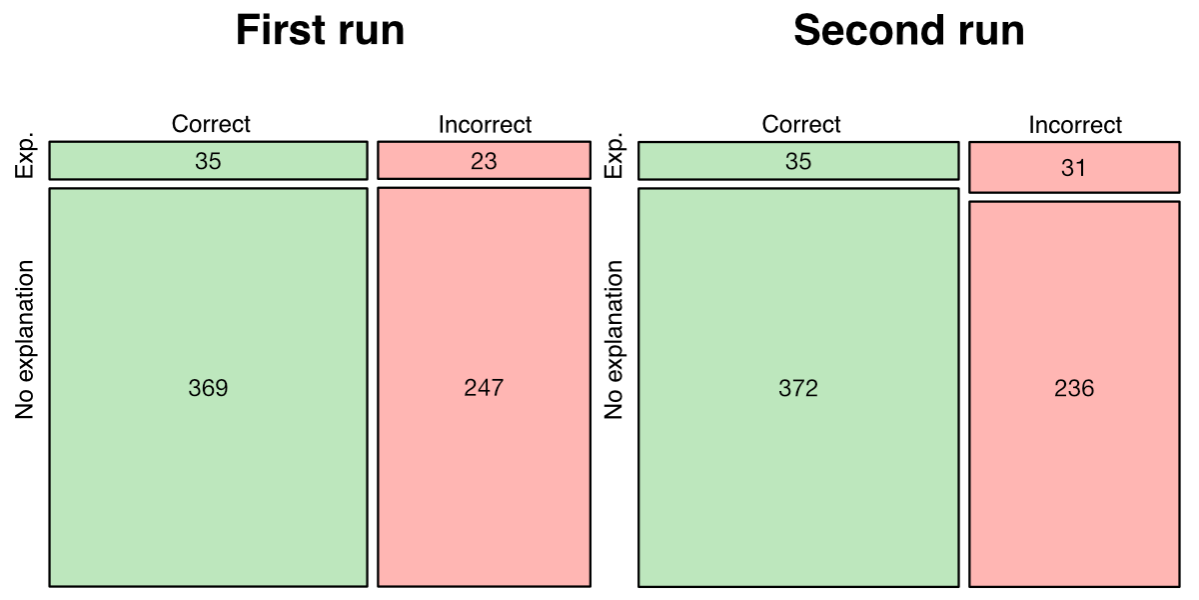
Mosaic plot depicting the relationship between ChatGPT’s proclivity to provide a novel explanation and answer accuracy. Exp.: explanation provided.

ChatGPT provided novel explanations in response to 58 (8.61%) questions in the first run and 66 (9.79%) questions in the second run. A novel explanation was provided in response to just 18 (2.67%) questions in both runs, illustrating significant stochasticity in the relationship between prompt and output. The proclivity of ChatGPT to provide a novel explanation had no bearing on accuracy in the first (Fisher exact test odds ratio 1.02, 95% CI 0.57-1.85, *P*>.99) or second (Fisher exact test odds ratio 0.72, 95% CI 0.42-1.24, *P*=.23) iterations (Figure 4).

## Discussion

This study makes 5 significant observations. First, performance in a national primary care examination cannot be passed by ChatGPT, although the platform came close in terms of accuracy to AKT pass marks in recent years. Contrary to some academic and media reports, AI cannot replace human doctors who remain indispensable within general practice. As ChatGPT attained sufficient performance to pass medical school examinations, its semantic knowledge base appears to lie between the minimum standards to graduate as a doctor and to qualify as a GP [5,16]. Second, ChatGPT’s performance is highly variable between subjects, suggesting that NLP applications must be deployed within highly specified roles to avoid compromising efficacy. Given the impressive performance of ChatGPT in certain subjects of the AKT, chatbots may be capable of providing useful input within narrowly defined portions of primary care.

Third, ChatGPT expresses uncertainty or technical limitation in a small minority of the cases in which it provides an incorrect answer. This limits the confidence patients and practitioners may place in chatbots’ answers, as there is no obvious way to determine the model’s uncertainty. This increases the risk of decisions based on inaccurate answers that occur too frequently to allow these applications to be deployed without supervision; this limits the current potential of this technology to automate health care processes. Additionally, use of ChatGPT as an educational tool in primary care is compromised by its frequent errors, which may not be noticed by learners. Fourth, the proclivity or ability of ChatGPT to provide novel explanations has no bearing on the accuracy of its responses, which remains inconsistent—the application frequently “hallucinates,” describing inaccurate information as lucidly as with correct facts. This compounds the issues regarding application of chatbots as decision support tools or educational assistants as discussed above. Lastly, the difficulty of subject categories based on GP trainee performance does not correlate with ChatGPT’s performance at the subject level—human perceptions or manifestations of complexity or difficulty cannot be translated to NLP models without validation.

This study comprehensively assesses the performance of ChatGPT across the domains of primary care assessed in the AKT, with a large sample size providing a realistic estimate of the application’s prospects were it to sit an official AKT paper. This provides valuable insight into NLP chatbots’ strengths and weaknesses as applied to general practice and facilitates research into model development and implementation based on data-driven conclusions. However, there were 2 limitations to this study. First, passing the AKT does not equate to demonstrating ability to perform as a GP; subsequent models with improved performance may or may not be appropriate for autonomous deployment. GPs’ knowledge and skills are tested in a variety of ways from medical school onward, with the AKT representing just one of many official assessments. Second, questions containing images or tables could not be inputted to ChatGPT, which may have affected our results. Emerging multimodal LLMs such as GPT-4 are compatible with all questions in the AKT, and our protocol provides a benchmark and methodology for trials of future models.

ChatGPT has garnered particular attention in recent months due to its performance in tasks previously considered completable by humans alone, such as passing medical school examinations such as the United States Medical Licensing Examination [5,16]. Other LLMs have exhibited similar achievements, such as FlanPaLM [17]. The ability of ChatGPT to accurately answer questions, provide useful advice, and triage based on clinical vignettes consistently exceeds that of a layperson [5,18]. However, the accuracy of computational models’ answers to medical questions is yet to exceed that of fully trained physicians, with findings in the present context of primary care being no exception [16,17]. When ChatGPT is used as a medical advice chatbot, advice seekers are only able to identify that the source of provided advice is computational 65% of the time [19]. It follows that health care providers must protect their patients from inaccurate information provided by this technology, as they are unable to differentiate between computational and human advice [19]. This requirement for oversight limits the potential of LLMs to meaningfully change practice, as performance equivalent to that of experts is the minimum standard to justify autonomous deployment: there must be confidence in the accuracy and trustworthiness of answers from these applications [20,21].

The excellent performance of ChatGPT in certain sections of the AKT indicates that deployment may be feasible within strictly bounded tasks. NLP chatbots may provide useful assistance to clinicians, but application as an autonomous decision maker is not currently justified by exhibited performance. Examples of potential uses include interpretation of objective data such as laboratory reports, triage (a fully automated conveyor model or with human management of edge cases), and semiautonomous completion of administrative tasks such as clinic notes, discharge summaries, and referral letters [21,22]. Further work is required to engineer models with supraexpert performance in any domain of primary care, which could justify deployment as an autonomous component of care provision [21]. Additionally, uncertainty indicators or contingency messages where the model is unable to answer with accuracy could improve confidence in the information provided and, therefore, safety [19,20]. Specific study is required to ensure that new tools reduce rather than increase workload for GPs [23-25]. As this technology continues to advance, individualistic care must not be sacrificed: general practice consulting involves long-term development of a therapeutic relationship between patients and physicians, and chatbots should not be allowed to change this dynamic into an impersonal, transactional arrangement [21,24]. Optimal management of patients’ issues is governed by patients’ wishes and circumstances in addition to the empirical evidence base.

Chatbots leveraging advanced NLP models are an exciting innovation with the potential to ameliorate staffing pressures that disproportionately affect deprived areas [11]. However, improvement in domain-specific tasks is required to enable this technology to make a meaningful contribution. Improvement is not a simple matter of increasing the size of the data set used to train these large language models. Larger models do not always exhibit superior performance in highly specialized tasks such as answering medical questions [26]. This is likely due to most available training material being irrelevant to medical tasks, as text is sourced from across the internet. While training may be improved by sourcing greater volumes of domain-specific text, development is complicated by restricted-access sensitive patients’ data, which likely comprises the largest unused source of information for large language models. Concerns regarding privacy and transparency of use currently limit the access of the largest NLP engineering companies to these data [27]. Alternative means of improving performance include fine-tuning by inputting a set of prompts or instructions to the model before it is deployed on a medical task. Fine-tuning has been shown to improve the performance of models beyond that of larger (but untuned) models, and fine-tuned LLMs are still state-of-the-art in terms of performance in medical questions, despite competition from ChatGPT, GPT-3.5, and GPT-4 [6,17,26,28]. It follows that similar tuning protocols may be applied to GPT-3.5 or ChatGPT to further optimize performance—this may be explored in backend development or by chatbot users experimenting with initial prompts before initiating a trial.

Effective applications must be rigorously trialed in the same context as the one they are intended to be deployed in the future [24,29]. As evidence supporting the integration of previously developed chatbots into primary care has suffered from poor reporting quality and high risk of bias, improved research practices are necessary to ensure that contemporary innovation fulfils its potential in terms of translated into impactful changes in clinical practice [30]. Validated NLP models may be more broadly applicable, such as within different language mediums, but revalidation and proper clinical governance are essential mechanisms to protect patients from harm [31]. As LLM-based chatbots have only recently begun to exhibit human or near-human ability to complete complicated tasks [3], a new set of evidence is about to be generated: this represents an opportunity to improve research practices to maximize the chance of innovative applications translating into impactful changes in clinical practice [22]. NLP technology may prove to be an integral part of a solution to the issues of staffing shortages, population growth, and health care inequities.

## Appendix

Multimedia Appendix 1 Exemplar questions and answers on the ChatGPT interface; mosaic plots stratifying performance by question source; and table stratifying performance by subject alongside the number of recommendations for improvement given by examiners based on human examination performance.

## Abbreviations

AI: artificial intelligence
AKT: Applied Knowledge Test
GP: general practitioner
GPT: Generative Pretrained Transformer
LLM: large language model
NLP: natural language processing
RCGP: Royal College of General Practitioners
