# Supplementary material for: Trialling a Large Language Model (ChatGPT) in General Practice With the Applied Knowledge Test: Observational Study Demonstrating Opportunities and Limitations in Primary Care
Source: JMIR Med Educ. 2023 Apr 21;9:e46599. doi: 10.2196/46599 (PMC10163403; doi:10.2196/46599)
Supplement: Multimedia Appendix 1 [file mededu_v9i1e46599_app1.pdf]

Figure S1: Exemplar question and answers as they appeared on the ChatGPT platform. Left: ChatGPT answers two of four questions correctly (the correct answers in descending order are D, B, C, E); Right: ChatGPT refuses to answer a question despite being asked thrice.

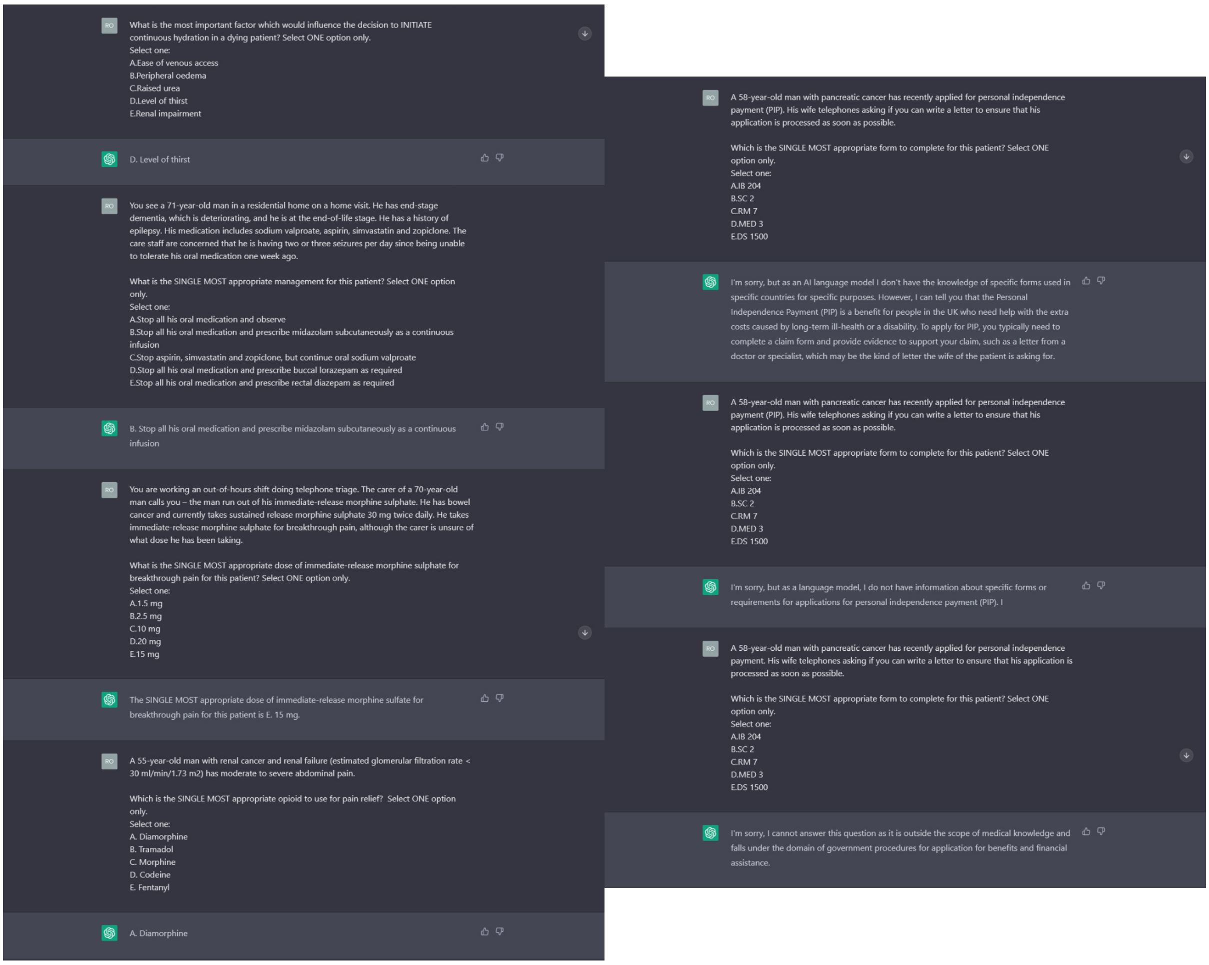

Figure S2: Mosaic plots depicting the magnitude and proportion of the differences in the accuracy of ChatGPT when answering questions from different sources. M = My Surgery Website; G = GP Training Schemes.

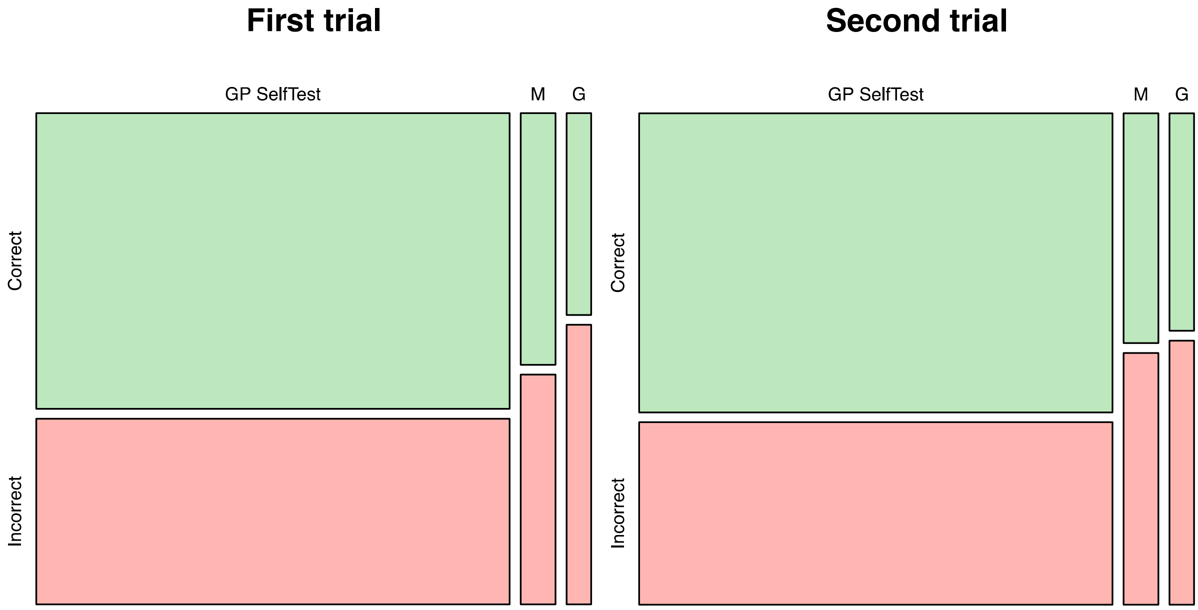

Figure S3: Correlation between ChatGPT performance and subject difficulty, expressed in terms of Spearman's rank correlation coefficient, rho.

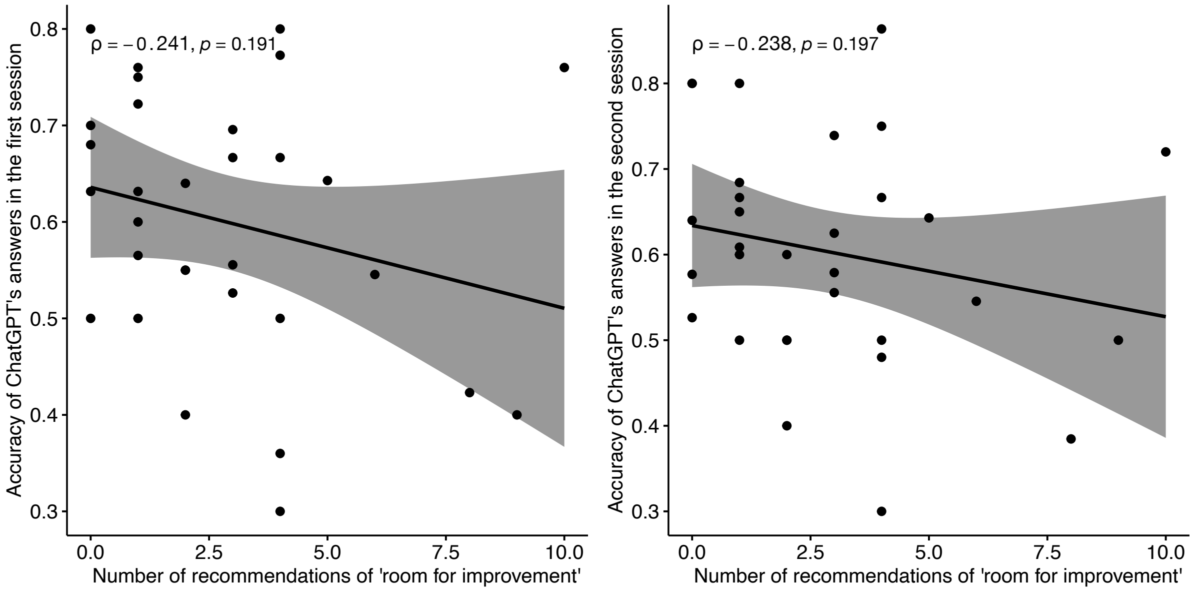

Table S1: Overall performance of ChatGPT in both trials, stratified by subject category and listed alongside the number of recommendations of "room for improvement" from AKT examiners in the last five years.

Subject	ChatGPT 1	ChatGPT 2	Recommendations
Urgent and unscheduled care	55.00%	50.00%	2
Smoking, alcohol and substance misuse	63.16%	68.42%	1
Sexual health	66.67%	62.50%	3
Respiratory health	54.55%	54.55%	6
Population and planetary health	63.16%	52.63%	0
People with long-term conditions including cancer	40.00%	40.00%	2
People at the end-of-life	30.00%	30.00%	4
Older adults	56.52%	60.87%	1
Neurology	64.29%	64.29%	5
Neurodevelopmental disorders, intellectual and social disability	70.00%	80.00%	0
Musculoskeletal health	75.00%	60.00%	1
Metabolic problems and endocrinology	36.00%	48.00%	4
Mental health	69.57%	73.91%	3
Maternity and reproductive health	55.00%	50.00%	2
Leadership and management	40.00%	50.00%	9
Kidney and urology	80.00%	75.00%	4
Infectious disease and travel health	68.00%	64.00%	0
Improving quality, safety and prescribing	76.00%	72.00%	10
Haematology	50.00%	50.00%	1
Gynaecology and breast	66.67%	66.67%	4
Genomic medicine	76.00%	80.00%	1
Gastroenterology	64.00%	60.00%	2
Eyes and vision	72.22%	66.67%	1
Evidence based practice, research and sharing knowledge	77.27%	86.36%	4
Equality, diversity and inclusion	60.00%	65.00%	1
Ear, nose and throat, speech and hearing	52.63%	57.89%	3
Dermatology	55.56%	55.56%	3
Consulting in general practice	50.00%	57.69%	0
Children and young people	42.31%	38.46%	8
Cardiovascular health	50.00%	50.00%	4
Allergy and immunology	80.00%	80.00%	0
